# The relation between sensation seeking, aggression and self-confidence with Needle stick and Sharp injuries among nurses

**DOI:** 10.1371/journal.pone.0330293

**Published:** 2025-09-04

**Authors:** Rana Ghasemi, Hossein Ebrahimi, Roya Najafi-Vosough, Shaban Ghasemi, Jamshid Rahimi, Rezvan Abedinloo

**Affiliations:** 1 Occupational Health Engineering Department, School of Public Health, Iran University of Medical Sciences, Tehran, Iran; 2 Occupational Health Research Center, Iran University of Medical Science (IUMS), Tehran, Iran; 3 Department of Biostatistics, School of Public Health, Hamadan University of Medical Sciences, Hamadan, Iran; 4 Iran University of Medical Sciences, Tehran, Iran; 5 Department of Occupational Health & Safety Engineering, Alborz University of Medical Sciences, School of Health, Alborz, Iran; 6 Student Research Committee, Hamadan University of Medical Sciences, Hamadan, Iran; Arak University of Medical Sciences, IRAN, ISLAMIC REPUBLIC OF

## Abstract

**Background:**

One of the most important occupational injuries experienced by nurses is needle sticks. The causes and factors of needle sticks are not fully known. This study was conducted with the aim of investigating the relationship between sensation-seeking, aggressiveness, and self-confidence with needle stick and Sharp injuries among nurses in a children’s and women’s hospital.

**Methods:**

This cross-sectional study was conducted on 143 nursing personnel of a children’s and women’s hospital in Iran. To collect data, people were asked to complete several questionnaires, including a demographic questionnaire, Arnett questionnaire of Sensation Seeking, aggression characteristics questionnaire (AGQ), and Rosenberg’s self-confidence questionnaire. They were also asked about the number of injuries caused by nurses’ needles and Sharp injuries in the last 12 months. Data analysis was done using SPSS software (version 22).

**Results:**

The results of this study showed, no relationship was between sensation seeking, aggression, and self-confidence with Needle sticks and Sharp injuries among nurses (P-value>0.05). Age and work experience have the inverse significant relationship with sensation seeking (P-value<0.05). The most common cause of needle sticks and sharp injuries was syringes (52.9%).

**Conclusions:**

There is no relationship between sensation seeking, aggression, and self-confidence with Needle sticks and Sharp injuries among nurses. Nurses with high age and more work experience show less sensation-seeking. Needle sticks and Sharp injuries in nurses were mostly caused by syringes.

## 1. Introduction

The safety of the workplace is never completely there in the healthcare industry due to the existence of various hazards [1]. in 2005, the health care and social assistance sector of the United States, is ranked second in Non-fatal occupational injuries (NIOSH 2009) [2]. getting needle sticks causes mental pressure and stress on the healthcare worker, because infectious diseases such as hepatitis B (HBV), hepatitis C (HCV), and human immunodeficiency virus (HIV) can easily be transmitted through Needle sticks and sharp injuries [3]. Needle stick is a common biohazard among healthcare workers in which a needle or sharp instrument contaminated with blood or other fluids pierces the skin and infects a person [4,5]. Sharp injuries, in health environments, unwanted and by mistake, are caused by sharp tools such as needles, lancets, scalpels, surgical tools, scissors, broken ampoules and other sharp tools, and cause wounds or cuts in the worker’s skin [6]. One of the most important occupational injuries experienced by nurses is needle sticks because, compared to other healthcare workers, nurses deal with injections and sharp objects, the blood and contaminated fluids of the patients due to the nature of their job [7]. The largest number of employees working in the health care system are nurses, with about 20 million nurses in worldwide [8].

The meta-analysis study showed that in Iranian hospitals, the prevalence of needle sticks among nurses was higher among other healthcare workers [5]. Like other incidents, various factors play a role in the occurrence of needle sticks, and one specific factor cannot be named in this case. A number of these factors such as job pressure, not having enough skills, ignoring necessary precautions, fatigue, unsuitable safety atmosphere, and others have been investigated by previous studies [9], but The causes and factors of needle sticks are not fully known. Until the accident reasons are not determined, it is not possible to take appropriate protective measures to prevent accidents [10]. The safety performance of nurses is a big challenge for healthcare environments due to the important tasks they carry, and the important factors affecting their safety performance should be investigated [11]. Human behavior depends on many factors including emotions, attitude, ethics, mood, personality, motivation, environment and other things. Any defect in any of these factors has a negative effect on human behavior and may cause danger and accidents [12].

Sensation-seeking is a personality trait in which a person attempts to have diverse, original, complex, and intense feelings and experiences and accepts all physical, social, legal, and financial risks [13]. Young adults who have high sensation-seeking scores tend to engage in risky activities and behaviors, Also, these people avoid working in a structure with a framework and low flexibility [14]. People with a high sensation-seeking tendency, because they consider less risk for new experiences, do activities more such as risky sex, reckless driving, smoking, drinking alcohol, and using illegal drugs [15].

Aggression is a behavior in which a person deliberately harms another person who is motivated to avoid harm (in the role of a victim) [16]. Aggression reduces driving safety. Aggressiveness causes risky driving behaviors, which may increase accident risk because drivers lost their concentration and vehicular control [17,18].

Having high self-confidence is one of the important personality traits in the nursing profession [19]. Nurses who have sufficient self-confidence have better control over their work and can manage complex clinical situations well [20]. Also, nursing students with high self-confidence are more successful in performing their clinical skills and experience less anxiety in difficult situations [19].

In any organization, workplace safety is an important yet challenging issue [2]. In the healthcare industry, nurses had the highest number of accidents [21]. In safety research in nursing, the variables affecting the safety performance of employees have been neglected [22]. Considering that about 80% of workplace accidents are related to the human factor, in this study we examined to the relation between sensation seeking, aggression characteristics and self-confidence with Needle stick and Sharp injuries of nurses in a children’s and women’s hospital.

## 2. Materials and methods

### 2.1. Participants

This cross-sectional study was conducted in a children’s and women’s hospital in Urmia, West Azerbaijan province. The recruitment period for this study began on 7-May-2022, and ended on 15-June-2022. It should be noted that the 250 nurses were working in this hospital. Based on the Cochran formula, the required sample size was calculated by 152 persons. The subjects were randomly selected from different sections. The exclusion criterion was the non-cooperation of the participants. Also, 9 questionnaires were incompletely completed and were excluded from the study. In total, the information of 143 participating nurses was entered into SPSS. Unfortunately, due to the non-cooperation of the nurses, we could not collect the rest of the questionnaires.

### 2.2. Data collection

Before starting the study, the necessary explanations about the objectives of this study and how to complete the questionnaires were explained to the participating nurses. The ethical approval code for conducting this study is from the Occupational Health Research Center of the Iran University of Medical Sciences IR.IUMS.REC.1400.410. In this study, the established ethical guidelines were followed. Participants signed the consent form. The questionnaires were collected after being distributed among the nurses.

### 2.3. Tools

In this study, 4 questionnaires were used, which include a demographic questionnaire, Arnett questionnaire of Sensation Seeking, AGQ aggression questionnaire, and Rosenberg’s self-confidence questionnaire. Each of these tools will be described in detail in the following sections.

#### 2.3.1. Demographic questionnaire.

The demographic questionnaire included general information such as age, sex, work history, marital status, and the name of the Sector where they work. Also, the participating nurses were asked about the number of needle sticks and cut injuries in the past year, and the types of devices that caused needle stick and cut injuries, in the form of self-report.

#### 2.3.2. Sensation seeking questionnaire.

Arnett’s Questionnaire consists of 20 items with a four-point Likert scale that are used to measure the amount of Sensation seeking in people. The minimum possible score will be 20 and the maximum score will be 100. A score between 20 and 40 is a low level of Sensation seeking. A score between 40 and 60 is average. A score higher than 60 is a high level of Sensation seeking. This questionnaire was evaluated in Iran by Rajabi in 2015 that the alpha coefficient obtained for this scale is 72% [23].

#### 2.3.3. Aggression questionnaire.

AGQ aggression questionnaire has thirty multiple-choice questions based on Likert type (never: 1, rarely: 2, sometimes: 3, and always: 4). The points of each question are added together. People who score equal to or more than 75 are classified as aggressive people. The normalization of this questionnaire was done by Zahedi Far and Najarian in Iran. The reliability of this questionnaire using Cronbach’s alpha method is 85%.

#### 2.3.4. Rosenberg self-confidence questionnaire.

Rosenberg’s self-confidence questionnaire has 10 items with a four-point Likert scale. Based on this, a score higher than 25 is considered good self-confidence, a score of 15–25 is regarded as moderate self-confidence, and a score less than 15 is considered low self-confidence. Based on the results of the study by Beshlideh et al., this questionnaire has satisfactory reliability coefficients (87% to 92%) and reliability.

### 2.4. Data analysis

First, the data was entered into SPSS software (version 22). In order to achieve the objectives of the study, at first, the normality of the data was investigated using the Kolmogorov-Smirnov or Shapiro-Wilk tests. To describe the data, descriptive methods including mean, standard deviation, frequency, and percent were used. Independent t-tests and Spearman’s correlation coefficient were used for mean comparisons. All the tests were done at a 95% confidence level.

## 3. Results

This cross-sectional study was conducted on 143 nurses of a children’s and women’s hospital in Urmia city in West Azerbaijan province. Among them, 51 nurses (about36%) experienced needle injury. About one-fifth of nurses had a Needle stick and Sharp injuries, 3 times or more. Also, the most common cause of needle sticks and sharp injuries was syringes (52.9%), peripheral vascular catheters (11.8%) and vein scalpels (11.8%) respectively. In addition, Table 1 shows the statistical distribution of demographic characteristics in two groups with and without needle and sharp injury. The results showed that mean values of age and work experience in subjects with needle and sharp injury were lower than those without needle and sharp injury.

**Table 1:** The statistical distribution of demographic characteristics of the nurses.

Variables	Needle stick and Sharp injuries	
	No N = 92	Yes N = 51
Age (Year)	37.05 (8.3)	32.65 (8.0)
Male	5 (5.4)	2 (3.9)
Female	87 (94.6)	49 (96.1)
Single	11 (12.0)	15 (29.4)
Married	77 (83.7)	35 (68.6)
Divorced	4 (4.3)	1 (2.0)
Infants	71 (77.2)	47 (92.2)
Women	17 (18.5)	3 (5.9)
Emergency	4 (4.3)	1 (2.0)
Work experience (Year)	12.84 (7.4)	8.37 (7.3)

Data are expressed as Mean (SD) or N (%). SD: Standard Deviation.

The values of the mean ± standard deviation of sensation-seeking, aggression, self-confidence and the number of Needle stick and Sharp injuries were48.07 ± 7.33, 50.39 ± 11.76, 21.56 ± 4.31 respectively. Table 2 reports the statistical distribution of the studied variables in two groups with and without the Needle stick and Sharp injuries experience.

**Table 2:** The statistical distribution of the studied variables.

Variables	Needle stick and Sharp injuries				P-value
	No (N = 92)		Yes (N = 51)		
	Mean	SD	Mean	SD	
Sensation-seeking	45.96	6.71	48.07	7.33	0.084
Aggression	50.60	12.50	50.39	11.76	0.919
Self-confidence	20.71	4.56	21.56	4.31	0.278

SD: Standard Deviation P-value P < 0.05.

The results of the independent t-test indicated that mean values of sensation-seeking, aggression, and self-confidence were no significantly different between the two groups with and without needle stick injuries (P > 0.05). The relationship between each of the variables of self-confidence, aggression, and sensation-seeking was investigated with age and work experience using Spearman’s correlation coefficient. The results showed that there is a negative significant relationship between age and work experience with sensation seeking, so with increasing age and work experience, sensation seeking decreases. Table 3 show these results.

**Table 3:** Relationship between age and work experience with sensation seeking.

Correlations		
**Variables**		**Sensation-seeking**
Age	Correlation Coefficient	−0.199
	P-value	0.020
Work Experience	Correlation Coefficient	−0.171
	P-value	0.042

P-value P < 0.05.

## 4. Discussion

Few studies have been done on the relationship between personality traits such as sensation- seeking, aggression, and self-confidence with needle stick and sharp injuries. In this study, Contrary to the assumptions presented, we did not find a relationship between sensation seeking, self-confidence, and aggression with needle sticks and sharp injuries. In past studies, women have been predicted to have lower levels of sensation-seeking and Disinhibition than men [24]. In the present study, women were more frequent than men (Table 1), and also, there was no relationship between gender and sensation seeking. Maybe it can be said, that this is why we could not find a relationship between sensation-seeking and Needle stick and Sharp injuries among nurses.

Unlike our study, the results of the study by Askari Majdabadi et al. in 2022 showed that needle sticking was more common among people who were aggressive and had high risk-taking and lower self-confidence [25]. This conflict may be due to the difference in the research environment, research tools and method of analysis. The research hospital in Askari Majdabadi’s study included various and extensive departments but in the present study, the research hospital was for children and women. In Askari Majdabadi’s study, path analysis was done using AMOS software, but in our study, Spss software was used. The size of the statistical population of Askari Majdabadi’s study was more than our study. For these reasons, this study may be in conflict with Askari Majdabadi’s study.

In this study, needle sticks and sharp injuries occurred mostly with syringes (52.9%), followed by peripheral vascular catheters (11.8%) and vein scalpels (11.8%). Also, the prevalence of needle sticks or sharps injuries was about 35% in the last 12 months. The rate of needle sticks and sharp injuries in our study (35%) is similar to the study of Adib HajBaghery from Iran (38.3%, 6 months) [26], Saadeh from Oman (39.7%, 6 years) [4], Salelkar from Goa, India (34.8%, 12 months) [27]. But the findings of the present study are more than the findings of Askarian from Iran (26.3%) [28] and Hope from Nigeria (23%) in the last 12 months, also in Hope’s study, most injuries happened while recapping needles and breaking injection ampoules [29].

One of the important findings of this study was that age and work experience have an inverse significant relationship with sensation seeking. Nurses with high age and more work experience show less sensation-seeking. This finding is consistent with previous studies that showed people with high age have low sensation-seeking [30,31]. It can be said maybe because people with a long work experience and High age prefer to work in a stable workplace with job security, they are less likely to be sensation-seeking.

In this study, women made up the largest number of personnel, and as a result, the rate of needle sticks and sharp injuries was high among them. Also, Nurses who had less work experience and lower age suffered more needle sticks and sharp injuries. In line with our study, a systematic review and meta-analysis study showed The factors such as female gender, young age, and less work experience, increase the chance of needle stick of healthcare personnel in Iran [32]. Ghanei Gheshlagh states in his study that the important reasons for the high prevalence of needle sticks among female nurses are the high number of female nurses compared to men, the high workload, and the low number of nurses compared to patients [5]. Also, a study in Malaysia showed that having more work experience, the incidence of needle sticks in them is lower. Also, the young age and inexperience of doctors may cause errors that increase the probability of needle sticks [33].

As one of the limitations of the current research, a limited population was investigated. It is suggested that in future studies, nurses working in different job positions and in different types of hospitals such as general, heart, burn, cancer and oncology hospitals and others should be taken into consideration. Also, the employees of medical and health centers, except for nurses, are made up of other people with different jobs who are exposed to needle sticks, such as doctors, and cleaners. It is suggested that these employees should be investigated in future studies.

## 5. Conclusion

Based on the obtained results, there is no relationship between sensation seeking, aggression, and self-confidence with Needle sticks and Sharp injuries among nurses. The results of this study showed that Nurses with high age and more work experience show less sensation-seeking. Also, needle sticks and sharp injuries were high among nurses with lower work experience and age.

Due to the fact that few studies have been done in this field, and also, the conducted studies also have contradictions, therefore, it is advised that researchers research more.

## Supporting information

S1 File Ghasemi. (SAV)
